# An inexpensive and easy-to-implement approach to a Quality Management System for an academic research lab

**DOI:** 10.12688/f1000research.24494.2

**Published:** 2020-08-06

**Authors:** Michael Hewera, Ann-Christin Nickel, Nina Knipprath, Sajjad Muhammad, Xiaolong Fan, Hans-Jakob Steiger, Daniel Hänggi, Ulf Dietrich Kahlert

**Affiliations:** 1 Clinic for Neurosurgery, Medical Faculty, Heinrich-Heine University Düsseldorf, Düsseldorf, 40225, Germany; 2 Center for Information and Media Technology, Heinrich-Heine University Düsseldorf, Düsseldorf, 40225, Germany; 3 Beijing Normal University, Beijing, China

**Keywords:** QMS, Quality Management, SOP, Automation, Lab book, electronic lab book, Cell banking, database

## Abstract

**Background:** Increasing concerns emerge regarding the limited success in reproducing data and translating research results into applications. This is a major problem for science, society and economy. Driven by industry or scientific networks, several attempts to combat this crisis are initiated. However, only few measures address the applicability and feasibility of implementation of actions into an academic research environment with limited resources.

**Methods:** Here we propose a strategy catalogue aiming for a quality management system suitable for many research labs, on the example of a cell culture focused laboratory. Our proposal is guided by its inexpensiveness and possibility of rapid installation.  For this we used eLabFTW, an electronic lab book, as hub for all other components of our Quality Management System (QMS) and digital storage of lab journals. We introduced Standard Operation Procedures (SOPs) as well as a managed bio bank for safer long-term storage of bio samples. Next, we set up a lab meeting as feedback mechanism for the QMS. Finally, we implemented an automated pipeline to be used for example for drug screens.

**Results**: With this effort we want to reduce individual differences in work techniques, to further improve the quality of our results. Although, just recently established, we can already observe positive outcomes in quality of experimental results, improvements in sample and data storage, stakeholder engagement and even promotion of new scientific discoveries.

**Conclusions:** We believe that our experiences can help to establish a road map to increase value and output of preclinical research in academic labs with limited budget and personnel.

## Background and Goal

By sharing our motivation and experiences in this effort, the aim of this article is serve as a reference supply for other leadership staff, both from academic or technical background, dedicated to responsible research innovation. Given the persistent hurdles in overcoming the bridge from biomedical lab discoveries to socio-economic applications in most of the projects, even with successful project development and breakthrough character, we dedicate this article especially to stakeholders involved in translational research. This spans from bench/animal scientists, to application specialists, to academic professors as well as to opinion leaders of funding agencies, scientific journals and governmental policy.

In our minds, laboratory quality is defined by highly accurate, efficient, transparent, data protective and through internal feedback algorithms constancy improving lab procedures. Laboratory quality control (QC) is the supervised sum of all measures put in place to aiming to achieve those parameters as most comprehensively as possible. We believe QC ensures lab operations streamlined to more efficiently than without leading to economic efficacy (by reducing time and resources due to minimizing wasteful replications), user loyalty and trust in own data since QC aims to minimize untrustable results. In a large picture, the authors believe that QC will shorten the delay of introducing innovation in clinics and market. Thus, unlike as considered by traditional society assumptions to restrict the innovation character of a lab, and, although for academic labs certain flexibilities need to be secured by not too heavy QC restrictions, QC represents an innovation mark and competition advancement especially in regards to sustainability (of results).

Two main factors urged us to introduce more stringent and monitored QC strategies in our lab. First, using same cell models and substance, a follow up project on an established, prior by at least two independent scientists of our lab confirmed model of pharmacological mediated suppression of a molecular signaling pathway in vitro, a new third lab member had difficult to replicate the model to the same extend. In the search of inequalities in the experimental design amongst the conducted, we identified differences in the manufacturer of the drug candidate, outdated cell authentication certificate, or even lack of knowledge of the genetic identity of one of the used cell model, as well as inconsistency in documenting the age of the cells when stressing them with the drug as contributors to the situation.

Due to its immense documentation load, the establishment of a quality management system (QMS) certified with ISO 9001 standard ^1^ in biomedical research labs, like ours (state-funded lab with less than 20 employees, only two of them being permanent positions, one being the lab head, the other a technician), is challenging. This is particularly true for academic labs where usually staff is changing rapidly and the progress for individual careers is often the dominator for operator’s decision making. However, it is shown, that a lack of quality management and transparency are two reasons for the ongoing reproducibility crisis ^2– 4^. We implemented a slim line QMS that should lead to an increase of confidence in our scientific lab outcomes by optimizing internal processes for elevated transparency and reproducibility.

## Methods

**Figure 1.**
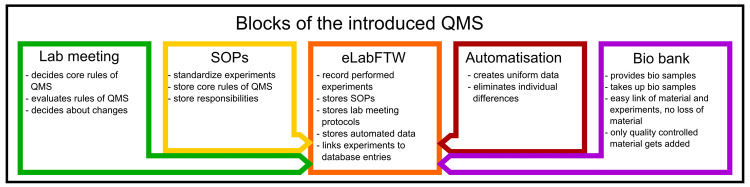
Blocks that form the QMS and their function inside of it. SOP: Standard operation procedure, QMS: Quality management system.

We introduced several technological tools to optimize processes, which combined, will form the QMS ( Figure 1) of our lab.

## Experience with freeware solution for electronic lab documentation

Transparent data management is a cornerstone for research integrity. As the basis of our work we set up an electronic documentation system to document all lab activities of all lab members. We chose eLabFTW ^5^ in the current version 3.4.8, an open source solution to track experiments with a powerful and flexible database. In the used version it provides all features necessary to provide the grounds of conformity with the principles of good research practices as proposed by the German research Foundation (DFG). This includes, not exclusively, the installation of a non-delete policy for created entries. The relevant software code can be found on GitHub platform ^6^. eLabFTW is made up from two parts. One is called ‘experiments’ and is the lab journal part of the software, the other is called ‘database’ and lets the user create text entries in self defined data types. The journal part of eLabFTW is used by each researcher to document their research, and for project management. Created entries cannot be deleted and changes to these entries will automatically be logged. The database part makes up the backbone of our QMS. We decided to share reading and search authorization for all entries amongst all registered personal accounts in our working group to increase transparency and to stimulate the critical interaction. Data storage, backup and archiving of eLabFTW data files can be easily implemented in regular executed local data management policy. All finished experiments are time stamped (digital signature), and blocked for editing from any user by the server itself. This will secure the written text and attached primary data (pictures, raw data from lab machines etc.) from later manipulations, as they are now unchangeable and undeletable.

After trying a few electronic lab book solutions, eLabFTW was chosen as the documentation solution for our lab, because is has some advantages over other electronic lab books, which include: A strong encryption and modern codebase (only ELN with A+ rating on Mozilla’s Observatory), RFC 3161 compliant timestamping of experiments it is community developed through volunteers (by scientists, for scientists) and compatible with all common browsers (also mobile). Furthermore it allows the import and export in common file formats and is translated into various languages. An instance of eLabFTW is hosted on out universities own servers, which ensures high availability.

## Establishment of coherent protocol and lab tool documentation system

SOPs are a main principle of most QMS. To our experience, even within one working unit, for one given experiment various self-developed, self-adapted protocols are in use at the same time. By implementing a binding SOP policy for all users, defining the accountability of the data to qualify for the use in external presentation such as in a scientific publication, we aim to enhance process coherence in order to increase intra-lab reproducibility success. As first step, a template for SOPs was created and its categorization and storage were implemented in the electronic lab documentation system followed by the establishment of a writing procedure for how to complete the template was developed. Any person can then write a draft of a new SOPs, but creation of the SOPs and QMS implementation are managed by selected authorized identity (in our hands a governmental- certified technical assistant to ensure conformation with regulation processes), to avoid duplicates or development of multiple parallel versions of one subjects. Approved, newly established SOPs then are categorized by name, a standardized alphanumerical identifier, and version number. Then all new SOPs get uploaded to the database of the electronic lab book. If applicable, SOPs are electronically linked with each other if their execution/specification depends on each other. In the last year 91 unique procedures and recipes have been standardized. 31 of them have been revised at least once, to either correct them, or adapt them to new findings or necessities. The SOPs are built that way, that one SOP describes one specific process. For the example of a qPCR, this would be one SOP for RNA extraction, one for cDNA synthesis, and one for the qPCR itself. The SOPs cover every area of lab work, from cell culture, over molecular methods and buffer preparation, to lab maintenance and data management. Every new employee will be sent a package of our most used methods and receipts as well as SOPs related to his proposed project, prior to their first day of work, so they have the chance to already get familiar with the methods in theory.

Moreover, virtual one-to-one project development meetings have been integrated in our e-documentation system by weekly, time-stamped progress reports of each lab member. Those are composed of structure items common for academic research projects featuring presentation of new results, problems experienced, suggestions of problem management and update on current literature. Leadership provides feedback on each parameter within a timely manner to secure up-to-date project management. Importantly, apart our open visibility of progress reports and feedback to all team members ensuring full transparency for all stakeholders, we have set up visibility-restricted sub projects. Those more restricted groups are intended to exchange work-in–progress documents between the individual user and the leadership, that have certain impact on the “outside-presentation” of the lab, such as presentations or manuscript drafts. We found this virtual project development-meeting platform enables efficient time management of the leadership and has been proven suitable to be conform with guidelines to minimize social contact, that have been stated by governments worldwide to fight the Covid-19 pandemic.

## Bio Bank

According to FAIR principles, when publishing research results, used material and created bio samples (tissue, cell pellets, DNA, RNA, plasmids, etc.) need to be held in stock to allow for future replications of the experiments performed or to share lab tools with the community. By establishing our lab bio bank, we aimed to provide a centralized storage facility for bio samples both for experimental and clinical research projects The bio bank consists of a temperature surveilled 500 L -80°C deep freezer, that stores about 400 cryo boxes and a 110 L liquid nitrogen tank, that stores 40 cryo boxes. The nitrogen tank is used to store cell culture cryo vials only. Other samples are stored in the freezer. Both, freezer and nitrogen tank, contain labelled boxes with designated positions for bio samples. Stored samples are characterized by a unique identifier. Long term preservation is supported by using freezing approved label technology and designation with printing instead of handwriting. The entire needed lab infrastructure to install our bio bank is a standard set up in each lab and does not require extra investments. Due to the anticipated large and constantly increasing number of bio samples included in the storage, we decided not to integrate the storage data into the database of eLabFTW but used a separate SQL database as the digital complement of the physical bio bank. Nevertheless, samples and experiments can be cross referenced in both platforms using the unique identifiers of the samples in the experiment section of eLabFTW, thereby securing complete and intertwined electronic documentation in our QMS. All lab members can search for samples in the digital database. However only three people have write access and can add and delete entries. Only those three people can access the physical bio bank, to add and remove samples. With this setup we aim to minimize sample loss and mix up, as well as ensure proper labelling of the samples.

## Quality assessment of cell models

As for many research labs, most of our projects fundament on *in vitro* experiments. For most of the cancer research projects, we perform our work by using the standard disease model type: the cancer cell line. Meanwhile paying particular attention to create the most-accurate-as-possible recapitulation of the pathophysiology of the disease by using 3D cultures, we furthermore surveil our technical quality of the applied biological test matrixes. This includes a) the confirmation of authentication through short tandem repeat analysis (STR) of cultured models every 6 months, b) the use of as-young-as-possible cell models (monitoring cell passage number); c) surveillance and - if necessary - clearance of mycoplasma contamination (PCR-based mycoplasma detection) and d) adherence to culture procedures as defined in SOPs. The STR analysis is outsourced to an in-house service. There Quantitation was performed using the QuantusTM Fluorometer (Promega) and the QuantiFluor® dsDNA Sample Kit (Promega) following manufacturer’s instructions. A multiplex PCR of 21 STR loci (PowerPlex® 21 System Kit, Promega) was performed in a total reaction volume of 12.5 µl with 0.5 ng template DNA. Thermal cycling conditions were followed as described by the manufacturer. Capillary electrophoretic separation was performed on the ABI Prism® Genetic Analyzer 3130 equipped with a 36 cm Capillary Array/POP-4 (Applied Biosystems, Darmstadt, Germany) following manufacturer’s instructions. Data acquisition and analysis was performed using the ABI Prism 3130 Collection software (Applied Biosystems) and GeneMapperID® v.3.2 software (Applied Biosystems, Darmstadt, Germany). For STR and mycoplasma test we invest less than 20 USD in total. We believe that the adherence to chronic execution of those simple methods is an economical, feasible and robust measure to sustainably reduce the creation of low-value cancer cell line data.

## Feedback and update communication platform

A monthly gathering of all participating and interested stakeholders secures the communication of updates and feedback mechanism of the QMS. Here also the state of the QMS is constantly revised by all lab members, and the further development is coordinated. The date and location for the meeting is circulated well in advance to ensure high frequency of participation of invitees. The feedback platform is open for all stakeholders independently of profession, profession hierarchy and amount of involvement in hands-on lab work. Each time a protocol of the meeting is generated in form of meeting minutes-like listing including specification of attendance. Although sometimes time consuming due to extent exchange of opposing opinions on topics, we hypothesize the impact of this outreach effort to involve all participants in the policy development extends further then the QMS. We believe such a platform increases the teamwork atmosphere and group belonging hereby increasing work atmosphere. We also think this is a valuable tool to fight occurrence of inequality and discrimination in our working group. The generated meeting minutes will be used to permanently alter the QMS to guarantee the highest possible quality. For that they will also be uploaded to the electronic lab book.

## Automation for lab work execution

Automation is a standard in industry labs and clinical diagnostics. We implemented a robotic liquid handling instrument (Beckman Coulter Biomek FxP) connected with automated plate reading for performing pharmacology testing on a cancer cell (Vargas-Toscano *et al*.) ^7^. We chose this method as our implementation trial since this is one of our most frequently used lab procedures. Our validation experiments based on manual repetition of the experiment proves that the functionality of our automation assay. Besides the accurate execution, the possibility of direct data reporting into the electronic lab documentation system and the possibility of electronic surveillance of executed pipetting for error reduction makes the inclusion of automation a valuable measure to increase reproducibly and transparency of our work. Given the existence of low-cost pipetting automation, more economically feasible options instead of the Biomek solution are available. Here, single-dose pumps in particular come to mind. These are of cause not as comfortable as robotic systems but fulfill the need of standardized pipetting. Used pumps can be bought for less than 1000 USD.

## Results and discussion

In our experience the approach of using an all-digital lab book with shared reading rights and automated storage of raw data, does not only guarantee data management according to FAIR ^8^ or increase the transparency according to the Hong Kong principles ^9^, but also helps to increase motivation of the team in general and thereby improving scientific development of the users. From all 17 lab members (at the time of publishing this), only 4 are not using the electronic lab book for recording their experiments. Those are members who are at the end of their projects and were therefore encouraged to keep their old method of recording, for consistency. Yet also those people use the database part of the electronic lab book, as well as the SOPs. The implementation of eLabFTW was a rather quick process. It is hosted on our university servers and as soon as the IT department set up a user group for our lab, our lab technician could populate the internal database during less than a week, as all inventory lists, primer lists, enzyme lists etc. where already available in a digital format and only had to be converted and imported to eLabFTW. As soon as this was done, the whole lab had a one day introduction into the functions of eLabFTW and where then able to use it. As eLabFTW is an open source freeware, there were no additional costs by our lab to use it. Exept from the 4 users mentioned before, for existing users old data is still stored in hand written lab books, but new projects are documented electronically. All new lab members will directly start documentation with the electronic lab book.

The use of SOPs, which were uploaded to the electronic lab book, lead to the standardization of particular processes in the lab. Before researchers performed several experiments with protocols from other or former labs. This leads to lack of reproducibility inside of the lab. Now a higher consistency of experimental results is given, as all researchers perform each experiment in only a single way, minimizing individual differences. Initial creation of SOPs was a very time costly process, as we had to decide which protocols to use to form a standard procedure. In addition, we decided, that only one person should write the final version of the SOPs, to keep constant wording and style. However other lab members handed in drafts and were involved in creating preliminary versions for specific SOPs. Now the addition of new SOPs is not very time consuming, as a draft will be created by the researcher who introduces a new method, after this method is refined, and the person responsible for creating SOPs will finalize them.

Compared to shared freezers used in the lab, central administration by only a few people highly reduces the loss of samples and mix-ups. Furthermore, the bio bank and its digital complement, with the possibility of linking it to the electronic lab book, make it very easy to find all samples used in all experiments. Since the Bio Bank is relatively freshly implemented, we cannot give results for the improvement of storage over longer periods of time yet. Until now the model of a digital copy of the bio bank, combined with limited access to the physical bank is holding up. All samples that got stored in the bio bank are still unmistakably findable. We argue that due to the QMS we fulfill the requirements for storage of data and samples, according to good scientific practice guidelines, as proposed by DFG ^10^. The SQL-Database was set up by our IT department. Before that the samples have already been moved to the biobank and all necessary information was already stored in an Excel-File that could then be imported to the SQL-Database. Because SQL Servers are also available as freeware, there are no additional costs, then running a Server for a lab that wants to implement such a database.

STR and regular mycoplasma testing lead to rejection of low quality cell cultures, ensuring higher significance of results derived from experiments with those cells ( Table 1 ^11^). Especially for metabolics or pharmacoproteomics experiments, a contamination with mycoplasma can lead to wrongful results. In our experience, even the most skillful and experienced cell culture scientists cannot avoid the introduction of unrecognized contamination. We found out that both, mycoplasma PCR and STR-Analysis are no big additional time costs. The PCR can be set up in about 5 minutes and then runs for 2.5 hours in which regular lab work can be continued. As the STR-Analysis is done by an in-house service, we just need to provide the DNA which can be extracted in about 30 minutes. With the introduced QMS, in our lab we identified cancer cell models have established subclones with different genetic background due to inter cell line contamination. Those subclones were designed as a different cell line instead of a subclone. The introduction of the QMS leads to big project adjustments and in some case to termination of experiments. We also put on hold a scheduled submission of a manuscript draft for publication due to cell line miss-identification.

**Table 1.** Short tandem repeat (STR) profile of contaminated brain cancer cell line mix assumed to be BTSC349 (mix), which got contaminated with BTSC268 cells. For comparison and a reference for the scientific community, STR profiles of parental lines are provided as well. STRs with mixes of both cell lines are marked. For original data as supplied by in-house service, see underlying data ^11^.

STR	BTSC349	BTSC268	Mix	STR	BTSC349	BTSC268	Mix
**D3S1358**	14, 16	14, 15	14, 15	**vWA**	17	14, 18	14, 18 (17)
**SD1S1656**	15	15.3	15.3	**D21S11**	29	29, 30	29, 30
**D6S1043**	7, 19	11, 15	11, 15 (7,19)	**D7S820**	10,11	9, 10	9, 10 (11)
**S13S317**	12	8, 11, 12	8, 11, 12	**D5S818**	12	11	11 (12)
**PENTA E**	17, 18	12	12 (17, 18)	**TPOX**	8	8, 12	8, 12
**D16S539**	11, 12	9, 13	9, 13 (11, 12)	**D8S1179**	13, 14	11, 13	11, 13 (14)
**DS18S51**	12, 14	12, 13	12, 13	**D12S391**	21	21, 24	21, 24
**D2S1338**	17, 18	23, 24	23, 24 (17)	**D19S433**	12, 15	14	14 (12, 15)
**CSF1PO**	11, 12	12	12 (11)	**FGA**	20, 25	21, 22	21, 22 (20)
**PENTA D**	11, 13	10, 14	10, 14 (11, 13)	**AMEL**	xy	x	x
**TH01**	9, 9.3	6, 8	6, 8 (9.3)				

The implemented communication system already proved its worth by leading to updated and refined versions of some SOPs. Given the deliverables of the meetings have a large influence on future lab procedures, we have experienced an increase in involvement and commitment of responsible lab members in this effort. A regular non-science oriented lab meeting creates an opportunity for easier expression of general matters thereby facilitating interpersonal communication amongst stakeholders. The virtual one-to-one project development report-feedback loops provides an opportunity to timely manage diverse research branches with moderate resources needed at each time.

As the technologically most advanced component of our QMS, we implemented an automated pipetting system in our lab routines. We believe that the robustness and transparency of the data generation, as well as the simplicity of its use in supporting repetitive work procedures are the reasons for high attractiveness to many lab users and that robotic assays increase the value and innovation of our work. As such, the application of the screening identified a repurpose of a FDA approved neurotransmitter drug to inhibit growth of brain tumor cells. Given the recent high-profile publications revealing the importance of neurotransmitter signaling for the biology of brain cancer ^12^, our QMS coincidently enabled us to contribute to current line of cancer research that we had not purposely addressed without it. Our initial results applying this industry-like assay on drug resistance testing on cell models are very encouraging. However, conclusive evaluations on whether data quality is improved requires further projects comparing manual pipetting-derived data with corresponding data retrieved from the robot tool. Such a project is currently underway.

## Summary

We present a rapid-to-implement, feedback approved method guide for initial steps to improve value of preclinical lab deliverables in a budget-restricted academic research environment. Given the increasing concerns on the value of preclinical research, we believe our activities are in line with current goals of funding authorities, academic self-governance and help to improve trust and recognition of science in society. We hypothesize that side effects of a QMS can also reduce inequality/discrimination amongst stakeholders.

## Data availability

### Underlying data

Zenodo: STR Analysis results BTSC 349, BTSC268 and mix of both. http://doi.org/10.5281/zenodo.3901446 ^11^

This project contains the following underlying data:

- 15-1-Wiss2020-01-16.fsa (BTSC 349 short tandem repeat analysis)
- 15-3-Wiss2020-01-16.fsa (BTSC268 short tandem repeat analysis)
- 15-4-Wiss2020-01-16.fsa (Mixed cell line short tandem repeat analysis)

Data are available under the terms of the Creative Commons Attribution 4.0 International license (CC-BY 4.0).
