# Supplementary material for: An inexpensive and easy-to-implement approach to a Quality Management System for an academic research lab
Source: F1000Res. 2020 Aug 6;9:660. Originally published 2020 Jun 30. [Version 2] doi: 10.12688/f1000research.24494.2 (PMC7385541; doi:10.12688/f1000research.24494.2)
Supplement: Peer review history

# Peer review: Reviewer response for version 2

**DOI:** 10.5256/f1000research.28462.r68943

**Published:** 2020-09-01

**Authors:** Olavo B. Amaral, Clarissa Carneiro

**Affiliations:** 1 Institute of Medical Biochemistry Leopoldo de Meis, Federal University of Rio de Janeiro, Rio de Janeiro, Brazil

The revisions addressed most of our concerns – in particular, the objective of the article is stated more clearly, and important details on the lab and procedures have been provided. However, we note that during the process of the revision a number of problems in English usage have appeared, and the manuscript would clearly benefit from a language revision to improve readability.  We have noted some of these issues below, but this is clearly not an exhaustive list.

 Abstract:

- “Although, just recently established…”. There should be no comma after ‘although’.

Background and goal:

- “the aim of this article is serve”. Should be “to serve”.
- “In our minds, laboratory quality is defined by highly accurate, efficient, transparent, data protective and through internal feedback algorithms constancy improving lab procedures” – this sentence is quite hard to understand and should be rephrased.
- “as most comprehensively”. Should be “as comprehensively”.
- “We believe QC ensures lab operations streamlined to more efficiently” – something seems to be off in this sentence.
- “Thus, unlike as considered by traditional society assumptions to restrict the innovation character of a lab, and, although for academic labs certain flexibilities need to be secured by not too heavy QC restrictions, QC represents an innovation mark and competition advancement especially in regards to sustainability (of results).” – This sentence is very hard to understand as well.
- “Two main factors urged us to introduce more stringent and monitored QC strategies in our lab. First, using same cell models and substance, a follow up project on an established, prior by at least two independent scientists of our lab confirmed model of pharmacological mediated suppression of a molecular signaling pathway in vitro, a new third lab member had difficult to replicate the model to the same extend.” – There are various errors in this sentence, which is also very hard to read.
- “progress for individual careers is often the dominator...” – I don’t think “dominator” is the right word here.

Methods:

- “RFC 3161 compliant timestamping of experiments it is community developed through volunteers (by scientists, for scientists) and compatible with all common browsers (also mobile).” – there seems to be a period missing between “experiments” and “it”.
- “The SOPs are built that way, that one SOP describes one specific process.”. Should be “are built in a way that...”
- “Importantly, apart our...”. Should be “apart from our...”
- “Both, freezer and nitrogen tank, contain labelled boxes”. There should be no commas here.
- “most of our projects fundament on *in vitro* experiments”. “Fundament” is not a verb.
- “Each time a protocol of the meeting...”. Should be “Every time”.
- “To extent exchange”. Perhaps “extensive exchange”?
- “Of cause not as comfortable...”. Should be “of course”.

Results and discussion:

- “introduction into the functions”… Should be “introduction to the functions”.
- “Exept from…”. Should be “Except for”
- “there are no additional costs, then running a Server from a lab…” – something seems odd here.
- “wrongful results”. Should be “wrong”.
- “miss-identification…”. Should be “misidentification”.

Besides these language issues, other minor concerns include:

- Should the meetings description be under the “documentation system” heading? Perhaps this issue deserves its own subheading.
- The detailed description of STR analysis methods seems out of place and excessive in a methods section that is much more generic in nature. Moreover, there seems to be no reason to use the past tense here (which is likely an artifact from copying and pasting the methods from somewhere else). If this is meant to be the methods for the results on Table 1, perhaps the authors might consider placing them in the legend or in the same repository as the data.
- I still don’t quite grasp how the meetings and QMS would reduce inequality and discrimination (something that is mentioned in the methods and in the last sentence of the summary). If you want to keep this statement, please explain why this is the case.
- The results section mentions that “now a higher consistency of experimental results is given”. As asked in our previous review, do the authors have data to support this statement? If not, it should be marked more clearly as speculative.

Is the work clearly and accurately presented and does it cite the current literature?

No

If applicable, is the statistical analysis and its interpretation appropriate?

Not applicable

Are all the source data underlying the results available to ensure full reproducibility?

No

Is the study design appropriate and is the work technically sound?

Yes

Are the conclusions drawn adequately supported by the results?

Partly

Are sufficient details of methods and analysis provided to allow replication by others?

Partly

Reviewer Expertise:

Metascience and behavioral neuroscience.

We confirm that we have read this submission and believe that we have an appropriate level of expertise to confirm that it is of an acceptable scientific standard, however we have significant reservations, as outlined above.

# Peer review: Reviewer response for version 2

**DOI:** 10.5256/f1000research.28462.r68944

**Published:** 2020-08-07

**Authors:** Jan Vollert

**Affiliations:** 1 Pain Research, Department of Surgery and Cancer, Imperial College London, London, UK

Happy with this revision.

Is the work clearly and accurately presented and does it cite the current literature?

Yes

If applicable, is the statistical analysis and its interpretation appropriate?

Not applicable

Are all the source data underlying the results available to ensure full reproducibility?

Yes

Is the study design appropriate and is the work technically sound?

Yes

Are the conclusions drawn adequately supported by the results?

Partly

Are sufficient details of methods and analysis provided to allow replication by others?

Yes

Reviewer Expertise:

Pain Research, Placebo, Improvement of Clinical and Preclinical Trial Design

I confirm that I have read this submission and believe that I have an appropriate level of expertise to confirm that it is of an acceptable scientific standard.

# Peer review: Reviewer response for version 1

**DOI:** 10.5256/f1000research.27020.r65762

**Published:** 2020-07-27

**Authors:** Olavo B. Amaral, Clarissa Carneiro

**Affiliations:** 1 Institute of Medical Biochemistry Leopoldo de Meis, Federal University of Rio de Janeiro, Rio de Janeiro, Brazil

Summary:

 The article describes an experience with a quality management system (QMS) within an academic basic biomedical research laboratory. The subject is relevant to the life sciences in general, as concerns with research quality are on the rise, and describing the development and implementation of a QMS within a lab is a worthy contribution; however, we believe the article would benefit from major changes in its structure to make its objectives, methods and results clearer.

 Main concerns:

- Who is the intended audience for the article? If this is meant to be a template or example to be followed by laboratory researchers in general, we believe that more information about the concept of QMS and the process of developing it are probably warranted. On the other hand, very specific information about cell culture automation and STR profiling, such as that presented on Table 1, is only of interested to a very specific audience. If the article aims to be a description of QMS in cell culture labs, that would be a valid goal as well, but in this case this topic should be covered in more detail, and the text in other sections should be changed in order to reflect this aim.
- What are the objectives of the article? If the goal is to describe the effects of the QMS system on some aspects of quality, more data is needed to adequately assess its impact. If the goal of the article is to present the development of the QMS, that is fine as well, but the methods and results sections should then be restructured to reflect that goal – in this case, the methods should explain the decision process leading to QMS implementation, while the results would present the system as it was implemented. In either case, a more detailed description of each block of the QMS is warranted, either as methods or as results, depending on the objective of the article.
- What is meant by quality and by reproducibility? Both terms can be read with many different interpretations, so including definitions would help to avoid misinterpretations of the goals of the article. Note that the QMS seems to address multiple facets of these concepts, so clarification might be necessary within each block of the QMS. In particular, most lab researchers are not familiar with the literature on quality management, so a more in-depth review of these concepts is needed in the introduction.

Title and abstract:

- Given the direct and practical content of the article, we suggest the title would be improved by removing the first part (i.e. “Measures to increase value of preclinical research”) and leaving only the second one.
- While the abbreviation QMS is defined in the abstract, we believe it should not be used in the title, in which it cannot be defined. Instead, we suggest using the unabbreviated form.
- The whole abstract could be shortened and made more direct, particularly in the Introduction and Methods section.
- If the system has already been implemented (as seems to be the case), why are some sentences in the future in the methods section of the abstract (e.g “These will be stored…”)?
- The abstract “Methods” subsection contains information not available elsewhere in the full text article (i.e. “Moreover, we restrict presentations of our actions on those for what we received a positive feedback by the users regarding its applicability”). Moreover, such feedback data is never presented in the article.
- The “Results” subsection does not seem to contain any result, irrespective of what objective is intended (as described above).

Introduction:

- If the article is targeted at a life sciences audience, however general or specific, we would not expect most of them to be familiarized with quality assessment vocabulary or literature. With that in mind, we suggest including an operational definition of QMS. Additionally, there has been some debate about its implementation in the basic academic research lab; thus, describing this context and reviewing the literature on the subject would also beneficial to the readers.
- On a more specific development of the previous point, we suggest including a brief description of your motivations for engaging in this endeavor. Was this a requirement by a superior instance, such as the institution or a funding agency? Was this a bottom-up initiative from the researchers? This could both (a) help to reach readers that could identify with the same issues but were not aware of them and (b) help to contextualize the adopted strategies in a broader landscape of previous experiences.
- Independently of the goal or targeted audience, for a case-study such as this one, it is vital that the introduction includes a description of the lab setting for which this QMS was developed. This includes the number of people and their positions in the lab (such as students, technicians, postdocs, etc), the group’s research interests or focus areas and the institutional environment (such as governmental vs. private funding, discovery-based vs. applied, etc). While some of these features can be inferred from other sections of the text, they should be presented upfront in the Introduction.
- The three references cited on “that a lack of quality management and transparency are two reasons for the ongoing reproducibility crisis” do not seem to fully support the sentence, as they do not directly mention quality management. That sentence should either be corrected or clarified to better represent the cited articles.

Methods:

- The issues raised as general comments should have a strong impact on the structure of the methods section. If the goal is to describe the creation of the QMS, the decision process to create each component of the system should be included here. If the goal was to assess the QMS itself, a more objective description of the QMS itself is warranted, but new sections on data collection would be required. In each subsection of the Methods, we found one or a combination of these strategies, which causes the section to lack coherence and makes the reader unsure about what the objectives actually were.
- From our understanding, Figure 1 is intended to be a summary of the QMS system. However, it does not completely correspond to what is described in the text. On one hand, the “lab meeting deciding core rules of QMS” shown in the first block of the figure is not described in the Methods section; on the other the “Quality of Cell Cultures” subsection is not reflected in the figure.
- In the description of standard operating procedures (SOPs), it is not clear for what processes they are being developed. Listing at least the most used SOPs would greatly improve our understanding of the strategy; one cannot understand for example, how specific a process is described in each of the 91 SOPs. Ideally, the authors should share some examples in the article, and perhaps include a template as a supplementary material.
- The description of one-to-one meetings probably deserves its own subheading and should not be under the SOP subsection.
- As mentioned previously, the quality of cell culture assessments subsection seems out of context and should only be included if the authors mean to reach a very specific audience (e.g. labs mainly working with cell cultures). If they mean to use cell culture as an example of the QMS implementation, we would suggest describing it in the Results section and relating each step of the process to the QMS blocks that are described in the methods.
- In the Feedback and Update section, there are three consecutive sentences with “hypothesize”, “believe” and “think” as verbs. That said, do the authors have data on the subjects touched upon? If so, they should present it – otherwise, they might be excessively speculating on the impact of their intervention.
- Also in this section, the link between the meetings and the claims of reduced inequality and discrimination is not clear. Please clarify.
- When describing automation, a result is mentioned: “i.e. the application of the screening identified a repurpose of a FDA approved neurotransmitter drug to inhibit growth of brain tumor cells…”. This does not belong in the Methods section and should be moved to the Results. Moreover, the causal link between automation and the mentioned results is not obvious and could be better explained by the authors.

Results and discussion:

- As mentioned above, the structural questions about the objective of the paper addressed in the General section of this report should have an important impact on the “Results and discussion” section of the article. In general, we found it to be better structured than the “Methods” section. In particular, we appreciated the description of some quantitative results and recognition of some limitations. That said, the section is still scarce on providing actual measures of impact of the QMS implementation – and, if this is meant to be the goal of the article, should include more data on the subject.
- In the first paragraph, the authors mention an increase in team motivation and improvement of scientific development of lab members, while the second paragraph states that “now a higher consistency of experimental results is given”. Do the authors have any data to base these statements on? If so, it should be shown (and details of this assessment should be included in the methods). If not, these conclusions might not be warranted.
- We suggest including more information when describing the implementation of the electronic laboratory notebook. For example, this particular product requires server installation, which might not be accessible to many biomedical researchers. Did you have assistance from an IT department of your institution? Moreover, how was the process for the 13 people who were able to completely migrate to this system? Did they have to stop their experimental schedules to implement all changes at once or was it gradual?
- The description of cell culture contaminations (Table 1) is very specific and not understandable for readers with no experience with STR profiling. If this is to be presented, it definitely needs a general description of how the results should be read and interpreted. That said, perhaps the best option would to leave this information out of the main text of the article and present it as supplementary material.
- To support the claims of this being a QMS for resource-restricted research groups, more information regarding the implementation cost of all blocks of the system is needed. Exemplifying the financial costs of the cell culture assessment assays is a welcome contribution; this, however, could be expanded in other parts of the QMS, not only in terms of financial costs but also of human resources and time requirement.
- In various points, but especially when discussing meetings (i.e. “a non-scientific oriented lab meeting creates an opportunity for easier expression of general matters…”), it would be useful to know whether the authors’ impression is indeed shared by all of the lab personnel. Do they have any kind of questionnaire/feedback data to know whether this opinion extends beyond the authors themselves?

Summary:

- We suggest toning down the conclusions presented here. Given the methods and results presented as they are, there is little data to support the claims of fast, easy or low-cost implementation of the QMS. To substantiate this claim, more details relating to cost, speed and implementation challenges should be provided in the previous section.
- The same recommendation holds for increased value of preclinical lab deliverables – unless the authors present more data on the impact of QMS implementation, this conclusion might not be warranted.
- Once more, it is not clear what the hypothesis of a reduction of inequalities (also mentioned in the methods) and discrimination is based on, as this does not seem to follow logically from QMS implementation.

References:

- In general, we do not believe the study provides an adequate reference list. It is insufficient to provide context to the methods used or to the discussion presented.
- Reference 4 has the wrong title and links to an unofficial source. It should be corrected to the publisher’s version.
- There is a typo in Reference 10: it should read OSF Preprints.
- Reference 12 is listed with two “publishers”: F1000 and Zenodo. From the link, we infer that Zenodo is the correct one.

Is the work clearly and accurately presented and does it cite the current literature?

No

If applicable, is the statistical analysis and its interpretation appropriate?

Not applicable

Are all the source data underlying the results available to ensure full reproducibility?

No

Is the study design appropriate and is the work technically sound?

Yes

Are the conclusions drawn adequately supported by the results?

Partly

Are sufficient details of methods and analysis provided to allow replication by others?

Partly

Reviewer Expertise:

Metascience and behavioral neuroscience.

We confirm that we have read this submission and believe that we have an appropriate level of expertise to confirm that it is of an acceptable scientific standard, however we have significant reservations, as outlined above.

# Response

**Published:** 2020-07-29

**Authors:** Michael Hewera

**Affiliations:** Universitätsklinikum Düsseldorf, Germany

Dear Clarissa Carneiro and Olavo B Amaral,

We thank you a lot for your very detailed review of our publication.

Most of your suggestions have been introduced in the revised version of the article.

Best regards,

Michael Hewera

# Peer review: Reviewer response for version 1

**DOI:** 10.5256/f1000research.27020.r65763

**Published:** 2020-07-27

**Authors:** Frank Miedema, Judith de Haan

**Affiliations:** 1 Utrecht University, Utrecht, The Netherlands; 2 University Medical Center Utrecht, Utrecht, The Netherlands; 3 Open Science Program, Utrecht University, Utrecht, The Netherlands

It is an interesting manuscript that shows that research groups can improve the quality of their experimental work without spending lots of money. It is great that researchers feel the need to improve their own practice, even if that does not immediately lead to more publications or more funding, but because it will improve the reproducibility of their work and contribute to the quality of the whole research field they are working in.

 Some suggestions for improvement: 

- The level of details in this paper vary a lot, on the one hand it is very detailed  (the amount of DNA and volume for the reaction to check cell line stability), but the level of information on the bio bank is very limited.
- It seems that the authors try to write a ‘case report’ about their own lab and at the same time try to give recommendations for other labs. It might be more valuable to make a clear distinction between those two goals. Maybe write it as a case report and then provide recommendations in the discussion part. Maybe distinguish the recommendations too, material wise recommendations (check cell lines, buy a pipet pump of ~1000 USD), (data ) storages recommendations and attitude recommendations (have regular lab meetings about quality, make sure all lab members use the same digital notebook and SOPs).

Is the work clearly and accurately presented and does it cite the current literature?

Partly

If applicable, is the statistical analysis and its interpretation appropriate?

Not applicable

Are all the source data underlying the results available to ensure full reproducibility?

Yes

Is the study design appropriate and is the work technically sound?

Yes

Are the conclusions drawn adequately supported by the results?

Yes

Are sufficient details of methods and analysis provided to allow replication by others?

Yes

Reviewer Expertise:

Human cellular immunology, virology.

We confirm that we have read this submission and believe that we have an appropriate level of expertise to confirm that it is of an acceptable scientific standard.

# Response

**Published:** 2020-07-29

**Authors:** Michael Hewera

**Affiliations:** Universitätsklinikum Düsseldorf, Germany

Dear Judith de Haan and Frank Miedema,

Thank you for reviewing our publication.

Following your suggestion, we adapted the level of detail, by adding some information to the bio bank part of the text.

Best regards,

Michael Hewera

# Peer review: Reviewer response for version 1

**DOI:** 10.5256/f1000research.27020.r65819

**Published:** 2020-07-17

**Authors:** Jan Vollert

**Affiliations:** 1 Pain Research, Department of Surgery and Cancer, Imperial College London, London, UK

The authors present the quality management system they have implemented in their lab and lay out specifics and improvements to their previous processes. The reader would benefit from more elaboration of the motivation that initiated the change, a better structure in before/after comparisons, and a primer in quantifying compliance and satisfaction of the users.

**Specific points:**

- “ *However, it is shown, that a lack of quality management and transparency are two reasons for the ongoing reproducibility crisis* *^2– 4^* *.* “

While I fully agree with this statement, I think the reader would benefit from some elaboration on this topic: why and how do QS and in particularly organised documentation, increase reproducibility?

- eLabFTW: it would be interesting to lead the reader through your decision process, as I assume other labs will consider similar options as you did. Why did you choose this one over the others?
- The central element of this manuscript – the reporting on some bits on the motivation of the researchers to finally approach this step would be interesting, which could also allow for a better before/after comparison. Which processes have improved by the introduction of the new system, where do the users experience early benefit, where are the users rather reluctant?

I appreciate that some information on the before is given in the results section, but think that a separate, earlier header on “before” would improve the structure.

- Similarly, the reporting of enthusiasm within the group is relatively vague – maybe you could even add a small anonymous survey within the group which bits are most and least liked. This can quantify the impact of the system at least a bit.
- Minor point: I am always at risk of doing this myself, but I would recommend refraining from using terms like “significant influence” as the word “significance” is so strongly tied to p-values in research papers.

Is the work clearly and accurately presented and does it cite the current literature?

Yes

If applicable, is the statistical analysis and its interpretation appropriate?

Not applicable

Are all the source data underlying the results available to ensure full reproducibility?

Yes

Is the study design appropriate and is the work technically sound?

Yes

Are the conclusions drawn adequately supported by the results?

Partly

Are sufficient details of methods and analysis provided to allow replication by others?

Yes

Reviewer Expertise:

Pain Research, Placebo, Improvement of Clinical and Preclinical Trial Design

I confirm that I have read this submission and believe that I have an appropriate level of expertise to confirm that it is of an acceptable scientific standard, however I have significant reservations, as outlined above.

# Response

**Published:** 2020-07-29

**Authors:** Michael Hewera

**Affiliations:** Universitätsklinikum Düsseldorf, Germany

Dear Jan Vollert,

Thank you a lot for your very quick response!

Following your advise, we added a short part about the process of choosing eLabFTW as out electronic lab book and changed the several "significants".

We really liked your idea of small surveys, but think it would have been only feasable, if we also did the same one before the installation of the QMS.

Inside of the team we do have our feedback meetings, to get these responses. Unfortunately we cannot publish our meeting minutes as data though.

Best regards,

Michael Hewera
